# Comparison of Rates of Central Line–Associated Bloodstream Infections in Patients With 1 vs 2 Central Venous Catheters

**DOI:** 10.1001/jamanetworkopen.2020.0396

**Published:** 2020-03-04

**Authors:** William C. Dube, Jesse T. Jacob, Ziduo Zheng, Yijian Huang, Chad Robichaux, James P. Steinberg, Scott K. Fridkin

**Affiliations:** 1 School of Medicine, Division of Infectious Diseases, Department of Medicine, Emory University, Atlanta, Georgia; 2 Rollins School of Public Health, Department of Biostatistics and Bioinformatics, Emory University, Atlanta, Georgia

## Abstract

**Importance:**

National Healthcare Safety Network methods for central line–associated bloodstream infection (CLABSI) surveillance do not account for potential additive risk for CLABSI associated with use of 2 central venous catheters (CVCs) at the same time (concurrent CVCs); facilities that serve patients requiring high acuity care with medically indicated concurrent CVC use likely disproportionally incur Centers for Medicare & Medicaid Services payment penalties for higher CLABSI rates.

**Objective:**

To quantify the risk for CLABSI associated with concurrent use of a second CVC.

**Design, Setting, and Participants:**

This retrospective cohort study included adult patients with 2 or more days with a CVC at 4 geographically separated general acute care hospitals in the Atlanta, Georgia, area that varied in size from 110 to 580 beds, from January 1, 2012, to December 31, 2017. Variables included clinical conditions, central line–days, and concurrent CVC use. Patients were propensity score–matched for likelihood of concurrence (limited to 2 CVCs), and conditional logistic regression modeling was performed to estimate the risk of CLABSI associated with concurrence. Episodes of CVC were categorized as low or high risk and single vs concurrent use to evaluate time to CLABSI with Cox proportional hazards regression models. Data were analyzed from January to June 2019.

**Exposures:**

Two CVCs present at the same time.

**Main Outcomes and Measures:**

Hospitalizations in which a patient developed a CLABSI, allowing estimation of patient risk for CLABSI and daily hazard for a CVC episode ending in CLABSI.

**Results:**

Among a total of 50 254 patients (median [interquartile range] age, 59 [45-69] years; 26 661 [53.1%] women), 64 575 CVCs were used and 647 CLABSIs were recorded. Concurrent CVC use was recorded in 6877 patients (13.7%); the most frequent indications for concurrent CVC use were nutrition (554 patients [14.1%]) or hemodialysis (1706 patients [43.4%]). In the propensity score–matched cohort, 74 of 3932 patients with concurrent CVC use (1.9%) developed CLABSI, compared with 81 of 7864 patients with single CVC use (1.0%). Having 2 CVCs for longer than two-thirds of a patient’s CVC use duration was associated with increased likelihood of developing a CLABSI, adjusting for central line–days and comorbidities (adjusted risk ratio, 1.62; 95% CI, 1.10-2.33; *P* = .001). In survival analysis adjusting for sex, receipt of chemotherapy or total parenteral nutrition, and facility, compared with a single CVC, the daily hazard for 2 low-risk CVCs was 1.78 (95% CI, 1.35-2.34; *P* < .001), while the daily hazard for 1 low-risk and 1 high-risk CVC was 1.80 (95% CI, 1.42-2.28; *P* < .001), and the daily hazard for 2 high-risk CVCs was 1.78 (95% CI, 1.14-2.77; *P* = .01).

**Conclusions and Relevance:**

These findings suggest that concurrent CVC use is associated with nearly 2-fold the risk of CLABSI compared with use of a single low-risk CVC. Performance metrics for CLABSI should change to account for variations of this intrinsic patient risk among facilities to reduce biased comparisons and resultant penalties applied to facilities that are caring for more patients with medically indicated concurrent CVC use.

## Key Points

**Question:**

Do current methods to measure performance of central line–associated bloodstream infection (CLABSI) prevention interventions adequately account for variations in patient risk associated with concurrent use of multiple central venous catheters (CVCs) that are medically indicated?

**Findings:**

In this cohort study of 50 254 patients at 4 hospitals, the risk for CLABSI associated with a second concurrent CVC was estimated to be approximately 80%, nearly 2-fold the risk of CLABSI for a patient with a single CVC.

**Meaning:**

This finding suggests that risk for CLABSI associated with a second CVC is significant and of large magnitude, justifying efforts to modify methods of performance measurement involving CLABSI prevention.

## Teaser

This cohort study examines rates of central line–associated bloodstream infection (CLABSI) among patients with single vs concurrent central venous catheters to determine variations in risk associated with use of multiple central venous catheters.

## Introduction

Although rare compared with other health care–associated infections, central line–associated bloodstream infections (CLABSIs) remain an important preventable health care–associated infection.^1^ These infections remain associated with significant mortality and can adversely affect patient care.^2^ The Centers for Disease Control and Prevention have established goals to try to eliminate CLABSI and promote the National Healthcare Safety Network (NHSN) as the standardized reporting infrastructure to accomplish this.^3,4^ Since January 2012, hospital reimbursement by the Centers for Medicare & Medicaid Services has depended on public reporting of CLABSI rates using NHSN methods. In these programs, CLABSI rates, reported as a standardized infection ratio, are included in the hospital-acquired condition score. Hospitals in the worst-performing quartile receive reductions in their reimbursement from Centers for Medicare & Medicaid Services.^5^

Given the financial stakes, attributes of performance metrics related to CLABSI should be evaluated to ensure that facilities are being compared fairly.^6^ To be effective, performance metrics should be reliable and objective.^7,8^ A comparative metric should account for differences between facilities regarding patient risk for infection that are host-related and unrelated to actual performance of infection prevention.^9,10^ Although efforts to improve the NHSN CLABSI performance metrics have focused on standardized case ascertainment and adjustment for comorbidities,^7,8,11,12,13^ we contend that current NHSN methods for quantifying the risk period (ie, the denominator as specified by NHSN protocols) is flawed; the denominator currently reflects the sum of person–time at risk for CLABSI as central line–days (CLDs) incompletely.

Current risk adjustment only accounts for the category of patient location (eg, surgical intensive care unit, medical intensive care unit), hospital size (ie, number of licensed beds), and medical school affiliation.^14^ The output of this model is then multiplied by the number of CLDs to determine the number of expected CLABSIs. Currently, NHSN defines CLDs as the sum of patients on each day at a specified time with any CVC present; for example, 1 patient with 2 CVCs on 1 day counts as 1 CLD.^15,16^ However, a patient requiring more than 1 CVC at the same time (ie, concurrent CVC use) is likely to be at greater risk of CLABSI than a patient requiring only a single CVC. If concurrent CVC use confers substantial additional risk for CLABSI, NHSN risk adjustment methods could be improved to allow more fair comparisons when calculating a performance metric among facilities caring for patients for whom concurrent CVC use is a medical necessity more often than their peer facilities.^17^ To evaluate the increased risk of a patient with more than 1 CVC, we used a multiyear, multihospital data set and 2 statistical approaches to define the risk associated with concurrent CVC use on CLABSI incidence.

## Methods

### Study Population, Data Source, and Design

Using a cohort design, we retrospectively analyzed inpatients admitted from January 1, 2012, to December 31, 2017, to any of 4 geographically separated acute care hospitals, varying in size from 110 to 580 beds, in Emory Healthcare (Atlanta, Georgia). The Emory Healthcare institutional review board approved the study under expedited review with a waiver of consent under 45 CFR.46.110 and 21 CFR 56.110 because it poses minimal risk and fits the regulatory category F[5] as set forth in the Federal Register. This study is reported following the Strengthening the Reporting of Observational Studies in Epidemiology (STROBE) reporting guideline.

Using the Emory Healthcare clinical data warehouse, we obtained CVC insertion and removal data and patient encounter data. These data were generated using electronic barcode tracking of insertion details and have been validated.^18^ Patients eligible for inclusion were adults aged 18 years or older admitted to inpatient care who had at least 1 CVC inserted for at least 2 days, with a length of stay of 50 days or fewer (95th percentile of patients), and who had 5 or fewer unique CVC insertions (eliminating outliers) during hospitalization. Emory Healthcare surveillance data for CLABSI reported to NHSN were linked to patient encounters. These CLABSI excluded infections categorized as mucosal barrier injury CLABSIs, as defined by NHSN. Encounter data included demographic characteristics and *International Classification of Diseases, Ninth Revision, Clinical Modification* (*ICD-9-CM*)^19^ discharge codes (allowing calculation of Charlson Comorbidity Index scores), orders for total parenteral nutrition (TPN), and chemotherapy.^20,21^

Key exposure variables were mapped into mutually exclusive categories based on historical risks for CLABSI outlined by Maki et al^22^: implanted devices (ie, ports), peripherally inserted CVCs (PICCs), hemodialysis CVCs, or temporary CVCs (eg, short-term tunneled or nontunneled, introducers, pulmonary artery catheter). The CLD (removal date − insertion date), and proportion of overlapping dates (ie, concurrence) were calculated. Episodes with concurrence were defined as those in which at least 2 CVCs were present on at least 2 of the same days. We created 2 variables for CLD: NHSN CLD (1 CLD for each day any CVCs were present), total CLD (counting each CVC on each day; eg, a patient with a port-a-cath for 3 days and a PICC for the same 3 days would contribute 6 days, instead of 3 days as in NHSN), and proportion of NHSN CLD with concurrence (1 − NHSN CLD / total CLD). For patients who developed a CLABSI, CLDs were counted only up to the date of CLABSI.

### Propensity Score Adjustment of Encounter-Level Data

In an attempt to reduce some of the bias inherent in evaluating the risk of a rare outcome, we selected a group of comparison patients without concurrent CVCs who were most comparable to patients with concurrent CVCs through propensity score adjustment of the cohort. This process used the *MatchIt* package in R statistical software version 3.5.0 (R Project for Statistical Computing) to determine a patient’s likelihood of having concurrent CVCs (using a dichotomous variable for any concurrence) given the set of covariates (ie, hemodialysis, chemotherapy, TPN, and Charlson Comorbidity Index score).^23,24^ Based on the likelihood of concurrence, we selected 2 patients without concurrent CVCs for each patient who had concurrent CVCs. We used this small cohort in a propensity score–adjusted model to control for all 4 factors and estimate the magnitude of risk associated with any concurrence during hospitalization.^25,26^

### Creating CVC Episode Data for Survival Analysis

A second analytic approach divided each eligible episode into distinct CVC episodes that included a single CVC (serial CVCs or a single CVC) or concurrent CVCs (the dates when 2 CVC were in place). A given CVC could contribute to a single CVC episode (limited to dates with single CVC) and a concurrent CVC episode (limited to dates with concurrent CVCs). Because concurrent CVC episodes could be many combinations of CVC types, to ensure more interpretable output, CVC type was categorized as lower risk (ie, PICCs, ports, or dialysis) or higher risk (ie, all other types) based on a previous systematic review.^21^ For any individual patient, a CLABSI was attributed to the CVC episode that occurred at the time or within 2 days of the CLABSI onset.

### Statistical Analysis

Initial descriptive analysis included all patients up to the time of the first CLABSI. When comparing patient characteristics among groups, we limited further analysis to those patients with either use of 1 CVC at a time (defined by any combination of CVCs with no concurrence; ie, placed sequentially without any overlap) or those with use of up to 2 CVCs at a time (patients with ≥3 CVCs used on a single date were excluded). This was done to minimize bias attributed to the third or fourth CVC. Statistical significance of the differences in groups were evaluated using χ^2^ tests for the categorical variables, 2-sample *t* test for the age variable, and Mann-Whitney *U* tests for the 3 CLD variables, as they were not normally distributed. Next, we completed a logistic regression model with a binomial distribution using our propensity score–adjusted data. The outcome of interest was the first occurrence of CLABSI. The risk factors included the propensity score, any CVC concurrence, high CVC concurrence (an indicator variable based on the median proportion of overlapping CVC use [ie, >two-thirds of NHSN CLD]), hospital, age, and sex. A second model used total CLDs instead of NHSN CLDs to illustrate reduction of risk when accounting for all CLDs. All analyses (unless otherwise noted), data management, and data cleaning were conducted in R statistical software version 3.5.0 (R Project for Statistical Computing), using the *lubridate* and *tidyverse* packages.^27,28^

Cox proportional hazards regression was used to model the time from CVC insertion to CLABSI, in which the type of CVC episode served as a time-dependent covariate. Forward stepwise model selection was performed in which all variables were considered for inclusion, and the significance level for variable entry and exit was set at 5%. The proportional hazards assumption was tested by including time interaction terms in the model and checking for significance. Hazard ratios (HRs), 95% CIs, and *P* values were calculated using SAS software version 9.4 (SAS Institute). Estimated survival curves were generated. *P* values were 2-sided, and statistical significance was set at .05. Data analysis was conducted from January 2019 to July 2019.

## Results

During the 4-year period, a total of 50 254 patients were admitted to 52 474 hospitalizations that included use of a CVC for at least 2 days. The median (interquartile range [IQR]) age was 59 (45-69) years, and 26 661 patients (53.1%) were women. In all, 64 757 CVCs were used. Concurrent CVC use was recorded in 6877 patients (13.7%). Reasons for CVC use included hemodialysis (8531 patients [17.0%]) and TPN (5702 patients [11.3%]), and the most frequent indications for concurrent CVC use were nutrition (554 patients [14.1%]) or hemodialysis (1706 patients [43.4%]) (Table 1). Overall, 647 patients (1.2%) developed CLABSIs; CLABSI occurred more often among men (358 patients [55.3%]), and CLABSIs were more common among patients with a single CVC (438 patients [67.7%]) than patients with 2 CVCs (79 patients [12.2%]) or patients with more than 2 concurrent CVCs (130 patients [20.1%]). Approximately one-third of patients with CLABSI (209 patients [32.4%]) had concurrent CVCs at some point in their hospital stay (Table 1). The largest of the 4 hospitals, hospital C, housed most of the patients with CLABSI (421 patients [65.1%]), although all hospitals were represented. Patients with CLABSI were rarely receiving chemotherapy (29 patients [4.5%]), but often were receiving TPN (35 patients [35.1%]) or hemodialysis (119 patients [18.3%]). Among patients with CLABSI with concurrent CVCs, PICCs (74 patients [14.3%]) or dialysis catheters (72 patients [12.0%]) were the most common types of catheters (Table 1).

**Table 1.** Characteristics of Patients With CVCs Stratified by Infection Status

Characteristic	No. (%)						
	Total (N = 50 254)	With CLABSI (n = 647)			Without CLABSI (n = 51 827)		
		>2 Concurrent CVCs (n = 130)^a^	2 Concurrent CVCs (n = 79)	No Concurrent CVC (n = 438)	>2 Concurrent CVCs (n = 2090)	2 Concurrent CVCs (n = 4578)	No Concurrent CVC (n = 45 159)
Hospital							
A	2196 (4.4)	3 (2.3)	2 (2.5)	7 (1.6)	37 (1.8)	107 (2.3)	2040 (4.5)
B	8201 (16.3)	12 (9.2)	2 (2.5)	31 (7.1)	363 (17.4)	628 (13.7)	7165 (15.9)
C	27 389 (54.5)	76 (58.5)	62 (78.5)	283 (64.6)	1166 (55.8)	2701 (59.0)	23 101 (51.2)
D	14 688 (29.2)	39 (30.0)	13 (16.5)	117 (26.7)	524 (25.1)	1142 (24.9)	12 853 (28.5)
Women	26 661 (53.1)	49 (37.7)	39 (49.4)	201 (45.9)	922 (44.1)	2236 (48.8)	23 214 (51.4)
Age, median (IQR), y	49 (45-69)	59 (43-69)	60 (46-68)	59 (45-67)	59 (47-69)	58 (47-67)	59 (45-69)
CCI score, median (IQR)	4 (2-7)	6 (4-8)	4 (2-7)	4 (2-7)	6 (4-8)	5 (3-7)	4 (2-7)
Receiving chemotherapy	1793 (3.6)	2 (1.5)	3 (3.8)	24 (5.5)	21 (1.0)	90 (2.0)	1653 (3.7)
Receiving TPN	5702 (11.3)	39 (30.0)	16 (20.3)	95 (21.7)	479 (22.9)	675 (14.7)	4398 (9.7)
Receiving hemodialysis	8531 (17.0)	42 (32.3)	30 (38.0)	47 (10.7)	901 (43.1)	1779 (38.9)	5732 (12.7)
CVC type							
Port	12 834 (25.5)	25 (19.2)	33 (41.8)	103 (23.5)	253 (12.1)	1149 (25.1)	11 271 (25.0)
PICC	19 092 (38.0)	46 (35.4)	28 (35.4)	128 (29.2)	731 (35.0)	2158 (47.1)	16 001 (35.4)
Other^b^	18 325 (36.5)	76 (58.5)	57 (72.2)	169 (38.6)	1649 (78.9)	3048 (66.6)	13 326 (29.5)

Abbreviations: CCI, Charlson Comorbidity Index; CLABSI, central line–associated bloodstream infection; CVC, central venous catheter; IQR, interquartile range; PICC, peripherally inserted central catheter; port, implanted central venous catheters requiring percutaneous puncture to access; TPN, total parenteral nutrition.

^a^ Concurrence was defined as any 2 CVCs present for 2 or more of the same days.

^b^ Includes CVC types not listed in the other categories (eg, pulmonary artery catheter, introducer, tunneled CVC, multilumen catheter).

The propensity score–adjusted data set included 11 796 patients, using their first encounter in the data set. Patients with concurrent CVCs were similar to the propensity score–matched control group in terms of hospital location, sex, age, TPN use, chemotherapy use, and Charlson Comorbidity Index score, but some differences in CVC types were present (Table 2). In this analysis, 155 patients (1.3%) developed CLABSI, including 74 of 3932 patients with concurrent CVCs (1.9%) and 81 of 7864 patients without concurrent CVCs (1.0%). The median (IQR) NHSN CLDs was 11.0 (6.0-18.0) CLDs for the group with concurrence and 5.0 (3.0-10.0) CLDs for the nonconcurrent group; while the median (IQR) of total CLDs was 17.0 (10.0-28.0) CLDs among the concurrent group, and unchanged for nonconcurrent group. The median (IQR) percentage of total CLD accounted for by NHSN methodology in the concurrent group was 66.7% (40.0%-93.8%) (Table 2). Among patients with CLABSI, the median (IQR) duration of CVC use was 17.3 (9.3-23.8) NHSN CLDs, whereas among patients without CLABSI, the median (IQR) duration was 7.0 (4.0-13.0) NHSN CLDs (Table 3).

**Table 2.** Characteristics of Patients in Propensity Score–Adjusted Cohort by CVC Concurrence Status

Characteristic	No. (%)			*P* Value^b^
	Total (N = 11 796)	CVC Concurrence (n = 3932)^a^	No CVC Concurrence (n = 7864)	
CLABSI	155 (1.3)	74 (1.9)	81 (1.0)	.001
Women	5873 (49.8)	1917 (48.8)	3956 (50.3)	.11
Age, median (IQR), y	59.0 (47-69)	58 (47-67)	59 (47-69)	<.001
CCI score, median (IQR)	5 (3-8)	5 (3-7)	5 (3-8)	.18
Receiving chemotherapy	241 (2.0)	84 (2.1)	157 (2.0)	.61
Receiving TPN	1404 (11.9)	554 (14.1)	850 (10.8)	<.001
Receiving hemodialysis	5118 (43.4)	1706 (43.4)	3412 (43.4)	.99
CVC type				
Port	2267 (19.2)	1097 (27.9)	1170 (14.9)	<.001
PICC	3545 (30.1)	1644 (41.8)	1901 (24.2)	<.001
Other^c^	4233 (35.9)	2551 (64.9)	1682 (21.4)	<.001
CLD, median (IQR)				
NHSN^d^	7.0 (4.0-13.0)	11.0 (6.0-18.0)	5.0 (3.0-10.0)	<.001
Total^e^	8.0 (4.0-16.0)	17.0 (10.0-28.0)	5.0 (3.0-10.0)	<.001
NHSN CLD with >1 CVC, median (IQR), %^f^	0 (0-40.0)	66.7 (40.0-93.8)	NA	<.001

Abbreviations: CCI, Charlson Comorbidity Index; CLABSI, central line–associated bloodstream infection; CLD, central line–day; CVC, central venous catheter; IQR, interquartile range; NA, not applicable; NHSN, National Health Care Safety Network; PICC, peripherally inserted central catheter; port, implanted central venous catheters requiring percutaneous puncture to access; TPN, total parenteral nutrition.

^a^ Concurrence was defined as any 2 CVCs present for 2 or more of the same days.

^b^ For categorical variables, *P* values were calculated using χ^2^ test; continuous variables with normal distribution, 2-sample *t* tests; continuous variables with nonnormal distribution, Wilcoxon signed rank tests.

^c^ Includes CVC types not listed in the other 3 categories (eg, pulmonary artery catheter, introducer, tunneled CVC, multilumen catheter).

^d^ Calculated as 1 day for each day any CVC are present (eg, a patient can contribute a maximum of 1 NHSN CLD per day, regardless of the number of CVCs present).

^e^ Calculated as each day for each CVC (eg, a patient with 2 CVCs for 2 days would have 4 total CLD).

^f^ Calculated as ([Total CLD / NHSN CLD] / NHSN CLD) × 100 (eg, a patient who had 2 CVCs for their entire CVC experience would have a value of 100%, while a patient who only had 1 CVC at any given time would have a value of 0%).

**Table 3.** Characteristics of Patients in Propensity Score–Adjusted Cohort by CLABSI Status

Characteristic	No (%)			RR (95% CI)	*P* Value
	Total (N = 11 796)	With CLABSI (n = 158)	Without CLABSI (n = 11 638)		
Concurrent CVCs^a^	3932 (33.3)	74 (46.8)	3858 (33.2)	1.83 (1.34-2.50)	
Women	5777 (49.0)	68 (43.0)	5709 (49.1)	0.92 (0.67-1.26)	
Age, median (IQR), y	59 (47-68)	60 (47-67)	59 (47-68)	NA	.77
CCI score, median (IQR)	5.0 (3.0-8.0)	4.0 (2.0-7.0)	5.0 (3.0-8.0)	NA	.25
Receiving chemotherapy	241 (2.0)	9 (5.7)	232 (2.0)	2.96 (1.53-5.72)	
Receiving TPN	1404 (11.9)	31 (19.6)	1373 (11.8)	1.81 (1.23-2.67)	
Receiving hemodialysis	5118 (43.4)	64 (40.5)	5054 (43.4)	0.89 (0.65-1.22)	
CVC type					
Port	2247 (19.0)	46 (29.1)	2201 (18.9)	1.75 (1.24-2.45)	
PICC	3643 (30.9)	40 (25.3)	3603 (31.0)	0.76 (0.53-1.08)	
Other^b^	4167 (35.3)	79 (50.0)	4088 (35.1)	1.83 (1.34-2.50)	
CLD, median (IQR)					
NHSN^c^	7.0 (4.0-13.0)	17.0 (9.3-23.8)	7.0 (4.0-13.0)	NA	<.001
Total^d^	8.0 (4.0-16.0)	21.0 (11.3-33.0)	8.0 (4.0-16.0)	NA	<.001
NHSN CLD with >1 CVC, median (IQR), %	0 (0-40.0)	0 (0-74.1)	0 (0-40.0)	NA	.001

Abbreviations: CCI, Charlson Comorbidity Index; CVC, central venous catheter; CLABSI, central line–associated bloodstream infection; CLD, central line–day; IQR, interquartile range; NA, not applicable; NHSN, National Healthcare Safety Network; PICC, peripherally inserted central catheter; port, port-a-cath; RR, risk ratio; TPN, total parenteral nutrition.

^a^ Concurrence was defined as any 2 CVCs present for 2 or more of the same days.

^b^ Includes CVC types not listed in the other categories (eg, pulmonary artery catheter, introducer, tunneled CVC, multilumen catheter).

^c^ Calculated as 1 day for each day any CVC are present (eg, a patient can contribute a maximum of 1 NHSN CLD per day, regardless of the number of CVCs present).

^d^ Calculated as each day for each CVC (eg, a patient with 2 CVCs for 2 days would have 4 total CLDs).

In multivariate modeling, patients with more concurrent CVC use (ie, concurrence >66% of NHSN CLDs) were at 62% higher risk of developing CLABSI than comparable patients without concurrent CVC use or less concurrent CVC use (adjusted risk ratio [aRR], 1.62 [95% CI, 1.10-2.33]; *P* = .001). Similarly, each additional NHSN CLD was associated with an 8% higher risk of CLABSI (aRR, 1.08 [95% CI, 1.07-1.10]; *P* < .001) (Table 4). However, using total CLDs instead of NHSN CLDs, the increased risk for the high concurrence group became nonsignificant (aRR, 0.73 [95% CI, 0.45-1.16]; *P* = .19), while the increased risk for total CLDs remained significant (aRR, 1.06 [95% CI, 1.05-1.07]; *P* < .001) (Table 4). Notably, considering CLABSI is a rare outcome, the odds ratios approximate aRRs.^29^ Hospital did not have a significant effect on risk of CLABSI in either propensity score–matched model.

**Table 4.** Multivariable Logistic Regression Output for CLABSI

Variable	Adjusted Risk Ratio (95% CI)	
	Model 1^a^	Model 2^b^
NHSN CLD with concurrence, %^c^		
0	1 [Reference]	1 [Reference]
≤66	0.70 (0.48-1.08)	0.58 (0.36-0.92)
>66	1.62 (1.10-2.33)	0.73 (0.45-1.16)
NHSN CLDs, per 1-unit increase^d^	1.08 (1.07-1.10)	NA
Total CLDs, per 1-unit increase^e^	NA	1.06 (1.05-1.07)
Age, per 1-y increase	1.00 (0.99-1.01)	1.00 (0.99-1.01)
Male sex	1.36 (0.99-1.89)	1.35 (0.98-1.86)
Propensity score^f^	1.23 (0.23-6.24)	1.25 (0.23-6.35)

Abbreviations: CLABSI, central line–associated bloodstream infection; CLD, central line–day; CVC, central venous catheter; NA, not applicable; NHSN, National Healthcare Safety Network.

^a^ Adjusted for age, sex, propensity score, NHSN CLD, and proportion of NHSN CLD with concurrence (categorized as 0%, ≤66%, or >66%).

^b^ Adjusted for age, sex, propensity score, total CLD, and proportion of NHSN CLD with concurrence (categorized as 0%, ≤66%, or >66%).

^c^ Concurrence was defined as any 2 CVCs present for 2 or more of the same days. Percentage of concurrence was calculated as ([total CLD / NHSN CLD] / NHSN CLD) × 100 (median [interquartile range] for patients with concurrent CVCs, 66.7% [40.0%-93.8%]).

^d^ Calculated as 1 day for each day any CVC is present (ie, a patient can contribute a maximum of 1 NHSN CLD per calendar day, regardless of the number of CVCs present).

^e^ Calculated as each day for each CVC (eg, a patient with 2 CVCs for for 2 days would be considered to have 4 total CLDs).

^f^ Calculated as the output of a logistic regression model in which the outcome is any concurrence of CLABSI; the risk factors include Charlson Comorbidity Index score and receipt of hemodialysis, chemotherapy, or total parenteral nutrition.

For the CVC survival analysis, 50 254 hospitalizations were divided into 57 642 CVC episodes associated with 526 CLABSIs. There were a total of 31 126 low-risk single CVC episodes, including 205 associated with CLABSIs, and 11 906 high-risk single CVC episodes, including 107 associated with CLABSIs. Among 14 610 concurrent CVC episodes, 4930 concurrent CVCs included 2 low-risk CVCs, including 72 associated with CLABSIs; 7937 concurrent CVCs with 1 low-risk and 1 high-risk CVC, including 120 associated with CLABSIs; and 1743 concurrent CVCs with 2 high-risk CVCs, including 22 associated with CLABSIs. Significant risk factors for CLABSI included male sex (HR for women, 0.83 [95% CI, 0.69-0.98]; *P* = .03), receipt of chemotherapy (HR, 2.41 [95% CI, 1.62-3.58]; *P* < .001), and receipt of TPN (HR, 1.30 [95% CI, 1.05-1.61]; *P* = .03). Admittance to hospital A was associated with lower risk of CLABSI (HR, 0.49 [95% CI, 0.34-0.70]; *P* < .001). Adjusting for these factors and compared with having a single low-risk CVC, the daily excess risk of CLABSI associated with concurrent CVC use was approximately 80%, regardless of CVC type (2 low-risk CVCs: HR, 1.78 [95% CI, 1.35-2.34]; *P* < .001; 1 low-risk and 1 high-risk CVC: HR, 1.80 [95% CI, 1.42-2.28]; *P* < .001; 2 high-risk CVCs: HR, 1.78 [95% CI, 1.14-2.77]; *P* = .01). The HR for a single high-risk CVC was 1.44 (95% CI, 1.13-1.84; *P* = .003). In this model, the hazard of developing CLABSI associated with a concurrent CVC episode was significantly higher than among single CVC episodes, regardless of CVC risk type, starting as soon as day 7 of the concurrent CVC insertion (Figure).

**Figure.**
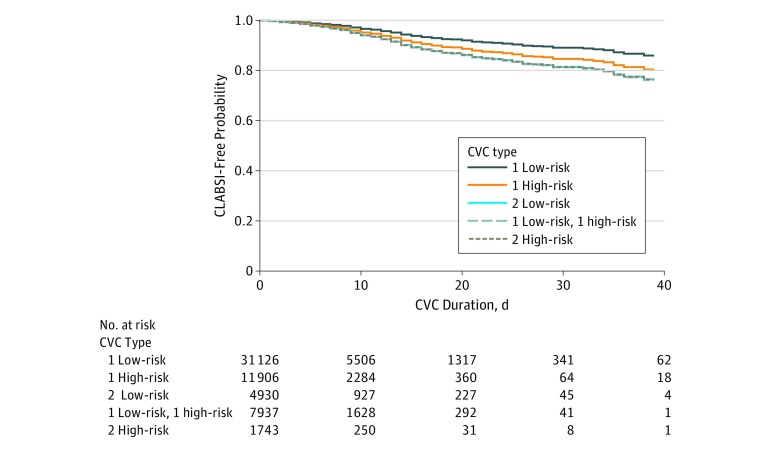
Time to Central Line–Associated Bloodstream Infection (CLABSI) Among Episodes of Central Venous Catheter (CVC) Use Stratified by Differing Combination of Concurrent Catheter Use Each line represents a hypothetical patient using 1 of the CVC scenarios accounting for facility, sex, and total parenteral nutrition and chemotherapy status.

## Discussion

This cohort study including more than 50 000 patients across 4 hospitals quantified an increase in risk for CLABSI between 60% to 80% associated with the use of concurrent CVCs. Although the exact magnitude of this risk varied depending on the statistical method used, it was consistently statistically significant and increased the risk of CLABSI nearly 2-fold. The implications of these findings are that the current performance metric using NHSN CLABSI rates does not adequately adjust for host risk factors, given the medical necessity of using 2 CVCs in some patients, such as dialysis and infusion of life-saving nutrition. We evaluated the excess risk at the patient level and at the CVC-episode level. As to the patient level, our finding that patient encounters in which the most of the CLDs occurred with 2 CVCs in place had a 62% increased risk of CLABSI suggests there may be a dose-response association or threshold of concurrent use at which the risk becomes significant. Our findings that the model adjusting for total CLDs rather than just NHSN CLDs demonstrated an elimination of this excess risk due to concurrence suggest that a potential solution to improved risk adjustment would be using total CLDs rather than current NHSN CLDs in risk adjustment by NHSN.

However, there are many subtle but important considerations in evaluating any excess risk, with the type of CVC potentially confounding this assessment, as certain CVC types are more likely to be used in concurrent CVC episodes and in patients who are more seriously ill. To better account for the different types of CVCs in use, our second analysis focused on quantifying the risk of an episode of CVC use associated with a CLABSI, accounting for the types of CVCs in use, and key risk factors for CLABSI. We observed that CVC episodes with 2 CVCs had a daily excess risk of CLABSI of approximately 80%. Our findings suggest that this begins at approximately day 7 of concurrent use. The excess risk was also observed comparing single CVC use of a high-risk CVC type (eg, temporary nontunneled subclavian CVC) to concurrent use of 2 low-risk CVC types (eg, dialysis port and PICC).^22,30^ If in fact each day of concurrent use increases the risk of a CLABSI nearly 2-fold compared with a single low risk CVC, regardless of concurrent CVC type, then accounting for this excess host risk for CLABSI is justified in use of CLABSI incidence as a performance metric. The NHSN method accounts for patient mix by comparing rates essentially between similar patient care locations; however, if similar locations have differing frequency of medically indicated concurrent CVC use, ignoring concurrent CVC use in measuring performance of CLABSI prevention will result in flawed measures of actual performance.

Attempts at estimating the increase in CLABSI risk due to CVC concurrence have been made by others.^15,31,32,33,34^ Our estimates are lower than those observed in single medical center study from 2014 by Concannon et al^33^ that quantified the attributable risk; however, the size of the study was smaller, and more importantly, it approached risk adjustment differently. Concannon et al^33^ used more traditional logistic regression adjusting for markers associated with disease severity. In contrast, for our first effort we used propensity score matching to define a group of control patients at similar risk to have concurrent CVC use. These differences together may be associated with the magnitude of the measured excess risk. Regardless, both studies document a significant and large excess risk. However, our study adds novel information by measuring this excess risk using CVC episode survival analysis quantifying the daily risk, controlling for patient mix and CVC type.

Other investigators have demonstrated the varied effects of counting total CLD would have on CLABSI rates.^15,34,35^ Based on these studies, the effect of comparing CLABSI rates using NHSN CLDs vs total CLDs appears to depend on the frequency of concurrent CVC use in the study population and the definition of concurrence (eg, any overlap at single time of day, at least several hours of overlap, entire days of overlap). Although simply counting all CLDs may be a simple approach to improving risk adjustment, it may not be the best approach. Studies describing the variability of concurrence among similar patient care locations (eg, medical and surgical combined units, surgical wards, surgical intensive care units) could be performed quickly to determine whether variability of medically indicated concurrent CVC use exists even between similar NHSN defined locations. For now, the evidence that the association of concurrent CVC use with CLABSI risk is significant, of high magnitude, and repeatedly observed seriously threatens the value of the existing methodology of NHSN CLABSI surveillance as a performance metric. Interinstitutional comparison using the NHSN benchmark disadvantages hospitals that specialize in caring for patients who require multiple medically justifiable CVCs concurrently. This may help explain why major academic medical centers are often the recipients of penalties associated with hospital-acquired conditions, including CLABSIs.^36^ While these results suggest that efforts are needed to improve risk-adjusted performance measurement, they do not negate importance of the efforts of health care practitioners that need to occur in tandem, such as reducing patient harm through removal of unnecessary CVCs. For example, about one-half of patients with 2 CVCs were receiving neither dialysis nor TPN, so exploration of increasing lumens rather than CVCs may be justified.

### Limitations

There are several limitations of this study. First, regarding interpretation of the findings, we assume in these 4 hospitals that there are medical indications for concurrent CVC use. Eliminating 1 of the concurrent CVCs in use when concurrent use is not medically indicated would be best the approach to CLABSI prevention and support current NHSN methods as appropriate for risk adjustment and performance measurement. However, concurrent CVC use is often medically indicated,^30^ and questioning the necessity of use only affects the interpretation of the findings, not the magnitude of the estimated risk. Although we did not perform a validation study to document medical necessity of concurrent CVC use in our cohort, our descriptive epidemiological examination suggests the patients with concurrent CVCs had medical indications for the second CVC: large proportions of concurrent CVC use occurred in patients receiving TPN or dialysis, and dedicated use of CVC for either of these procedures is considered standard of care.^22,30^ Other limitations include the potential for misclassification of CVC types or insertion and removal dates. These data were extracted from a clinical data warehouse that incorporated electronically captured data. Although there is potential for systematic errors in such electronically captured data, extensive validation of this system has been performed and reported on elsewhere.^18^ We also remained agnostic to site of insertion, as these data were not reliably available in the data warehouse. Additional limitations may include use of a conservative definition of concurrence: we required at least 2 days of overlap, and some patients with some hours or 1 day of overlapping CVC use would be considered single-line CVC episodes. However, misclassification in this direction would have biased our results toward the null. In addition, short-term dialysis catheters have been observed to be associated with a relatively high risk of CLABSI, while we categorized all dialysis catheters as low-risk CVCs in the survival analysis.^37^ We did this to allow more interpretable model results; however, any systematic misclassification of risk in this direction would have biased our results toward the null as well. Therefore, we are confident in the interpretation of our findings, even if the magnitude of the association may be slightly underestimated owing to this potential misclassification. Next, 2 of the study hospitals serve large oncologic, transplant, and hemodialysis populations, which may limit the external validity of our findings; however, the association was significant in the survival analysis after adjusting for the facility effect of the large community hospital, hospital A, which had implemented dedicated CVC insertion and maintenance teams early in the study.

## Conclusions

This cohort study provides large-scale, robust evidence of a large and significant excess risk for CLABSI associated with use of a second CVC concurrently with an initial CVC. The excess risk is nearly 2-fold that of a single CVC, an increased risk of approximately 80%; this finding adds to other evidence that accounting for total CVC use would produce a more accurate metric of CLABSI prevention efforts than the current NHSN method, which is agnostic to use of 2 CVCs at the same time. The best operational approach to account for concurrent use of medically indicated CVCs should be determined and implemented to improve the value of NHSN CLABSI reporting as a fair and meaningful performance metric.
